# Mechanism of the Immediate Effect of Balloon Dilation Therapy in Spinal Muscular Atrophy With Dysphagia: A High-Resolution Manometric Study

**DOI:** 10.7759/cureus.62191

**Published:** 2024-06-11

**Authors:** Mika Ogawa, Kunieda Kenjiro, Tomohisa Ohno, Keishi Okamoto, Ichiro Fujishima

**Affiliations:** 1 Department of Rehabilitation Medicine, Hamamatsu City Rehabilitation Hospital, Hamamatsu, JPN; 2 Department of Neurology, Gifu University Graduate School of Medicine, Gifu, JPN; 3 Department of Dentistry, Hamamatsu City Rehabilitation Hospital, Hamamatsu, JPN; 4 Department of Rehabilitation, Hamamatsu City Rehabilitation Hospital, Hamamatsu, JPN

**Keywords:** swallowing rehabilitation, balloon dilation therapy, cricopharyngeal muscle, high-resolution manometry, upper esophageal sphincter, pharyngeal function, pressure, dysphagia

## Abstract

Balloon dilation therapy (BDT) is used to treat pharyngeal dysphagia in patients with impaired upper esophageal sphincter (UES) relaxation due to cricopharyngeal dysfunction. However, the mechanism underlying this immediate effect remains unclear. Here, we present a case in which we investigated the immediate effects of BDT on UES dysfunction using high-resolution manometry (HRM). A 67-year-old man was diagnosed with spinal muscular atrophy (SMA). He gradually developed dysphagia, and a gastrostomy was performed. Despite continuing oral intake of supplemental nutrition, the patient developed dysphagia. Videofluoroscopic (VF) examination of swallowing revealed pharyngeal residue, while HRM showed weak pharyngeal contractility and impaired UES opening. BDT was performed to address the UES dysfunction. Immediately following BDT, VF demonstrated improved pharyngeal bolus passage. As for the UES function during swallowing, HRM revealed that the UES relaxation duration was significantly longer and the UES nadir pressure was significantly decreased. The patient continued the BDT before oral intake. HRM revealed immediate and prolonged UES opening with decreased UES pressure during swallowing as an immediate effect of BDT. This suggests that these findings represent the mechanisms underlying dysphagia in this patient with SMA. BDT resulted in an immediate improvement in UES function, potentially leading to dysphagia improvement. BDT should be combined with conventional rehabilitation for impaired UES opening. However, further studies are needed to confirm the long-term effectiveness of BDT for dysphagia due to SMA.

## Introduction

The upper esophageal sphincter (UES) opens during swallowing to enable the pharyngeal passage of the bolus. The cricopharyngeal muscle and the caudal portion of the inferior pharyngeal constrictor muscle are reported to be the muscular components of the UES [1]. Cricopharyngeal dysfunction (CPD) caused by neuromuscular diseases is a serious cause of dysphagia [1-4].

Balloon dilation therapy (BDT) is a safe and effective treatment for failed UES relaxation caused by CPD in patients [5-12]. Several reports have evaluated the immediate effects of BDT for dysphagia patients with failed UES relaxation using a videofluoroscopic examination of swallowing (VF) [11,12]. Additionally, BDT has shown clinical effectiveness when administered just before oral intake training with food or meals. The immediate effect of the BDT prior to oral intake improves pharyngeal bolus passage, avoiding pharyngeal residues and aspiration. However, no studies have investigated the mechanism of the immediate effect of BDT, focusing on pressure dynamics. Here, we report a case of spinal muscular atrophy (SMA) in which we investigated the immediate effect of BDT on UES dysfunction using high-resolution manometry (HRM).

## Case presentation

A 67-year-old male presented with dysphagia. The patient was clinically diagnosed with SMA at 60 years of age. He reported experiencing progressive dysphagia for approximately 10 years, accompanied by weight loss. His cognitive function was preserved. Tongue movement and mastication did not present any issues that would prevent ingestion. The saliva pooled in the pharynx was occasionally expectorated. He required assistance with activities of daily living due to muscle weakness throughout his body, including his extremities. However, he was able to eat independently without assistance. He underwent percutaneous endoscopic gastrostomy (PEG) at 62 years of age. He exhibited severe dysphagia classified as level 2 using the Food Intake LEVEL Scale (FILS) (swallowing training without food was performed) [13]. After undergoing PEG placement, aiming to improve his oral intake, the patient remained hospitalized for swallowing rehabilitation (e.g., forehead exercise for suprahyoid muscles [14], breathing training, and lingual resistance exercise [15]). Additionally, BDT with a small ball balloon was performed. However, pharyngeal residue in the pyriform sinus was not cleared sufficiently. Subsequently, the rehabilitation doctor introduced BDT with the double-balloon catheter (Create Medic, Yokohama, Japan). This catheter, consisting of a small inner ball balloon and a cylinder-type outer balloon, was used for sufficient dilatation of the UES. The dilatation time of the UES using the double-balloon catheter was approximately 3 minutes, once or twice a day. After five months of swallowing rehabilitation including BDT, his swallowing function gradually improved. His swallowing function progressed to FILS level 5, allowing him to consume easy-to-swallow foods orally in one to two meals, though supplemented nutrition continues. After discharge from the hospital, he attended the outpatient department for dysphagia treatment, including BDT. He continued BDT with a small balloon before each meal. Since performing BDT with the double-balloon catheter was difficult for the patient himself, the rehabilitation doctor administered it during his visits to the outpatient department. His oral intake remained stable, and the frequency of BDT with the double-balloon catheter was gradually reduced.

Approximately four years after initiating BDT, a follow-up assessment of the swallowing function was performed. Before BDT, a videoendoscopic examination of swallowing revealed weakened pharyngeal contractions and saliva pooling in the pyriform sinus. The VF revealed a bolus residue in the pyriform sinus due to weak pharyngeal contractility and impaired UES opening. Sliced jelly and thickened liquid were used. VF showed the laryngeal elevation was weak, and the swallowing reflex was delayed. He swallowed with spontaneous coughing occasionally to avoid aspiration of pharyngeal residues. Both BDT using double balloons and HRM were conducted during the VF examination. HRM was performed before BDT and post-therapeutic HRM was conducted immediately after BDT. We obtained written informed consent from the patient for publication of this report.

Balloon dilatation therapy (BDT) with the double-balloon catheter

Under fluoroscopic guidance, a double-balloon catheter (Create Medic, Yokohama, Japan) was gently inserted through the oral cavity until the balloon was estimated to be under the lower margin of the UES. Patients were instructed to phonate “Oh” to facilitate the insertion of the catheter from the oral cavity into the pharynx. Subsequently, 7 ml of air was injected into the balloon. After the balloon was pulled and the inner balloon was caught on the lower edge of the UES, 12 ml of air was injected into the outer balloon to continuously dilate the UES for 3 min. The external balloon of the catheter was expanded to a diameter of approximately 25 mm and a length of 42 mm. Sensation in the pharynx was present; however, the UES could be continuously dilated using the BDT without triggering a gag reflex.

**Figure 1:**
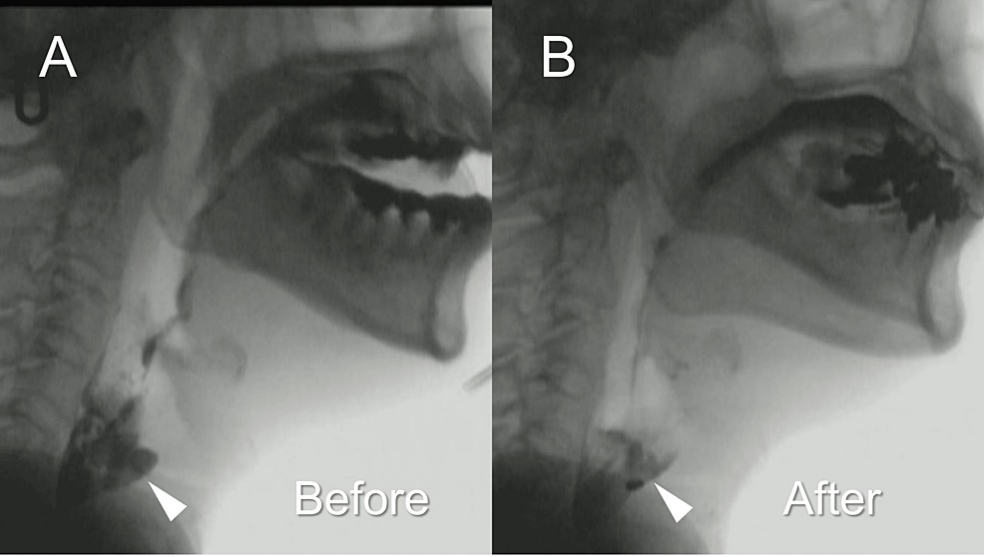
VF of the patient VF of the patient in a lateral view before and after BDT. Fluoroscopic images before (A) and after (B) BDT. (A) The pharyngeal residue in the pyriform sinus (arrowhead). (B) After BDT, the pharyngeal residue in the pyriform sinus is reduced. VF, videofluoroscopic examination of swallowing; BDT, balloon dilatation therapy

Post-BDT VF revealed improved pharyngeal bolus passage and decreased pharyngeal residue compared to baseline (Figure 1). Additionally, the patient reported improvement in dysphagia symptoms like residual pharyngeal sensation and swallowing difficulties. BDT was continued before meals with the same amount of air injected into the balloon.

High-resolution manometry (HRM)

**Figure 2:**
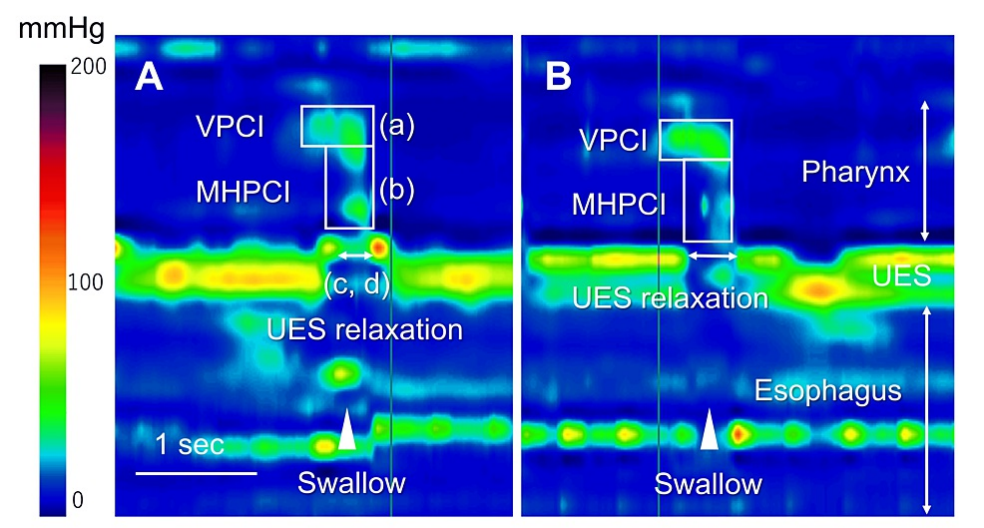
Pressure topography of the present case of a patient with dysphagia before (A) Before and (B) after balloon dilatation therapy. Spatiotemporal plots of liquid swallows. Y-axis, catheter position; x-axis, time. Pressure is indicated by the color scale: black or red for high pressure and blue for low pressure. VPCI and MHPCI were measured at the velopharynx (a) and meso-hypo pharynx (b), respectively. UES relaxation duration and UES nadir pressure were evaluated at (c, d). VPCI, velopharyngeal contractile integral; MHPCI, meso-hypopharyngeal contractile integral; UES, upper esophageal sphincter.

The swallowing pressure along the pharynx and UES was measured using an HRM (Unisensor AG, Attikon, Switzerland). HRM assessed five 3-mL swallows of thickened liquid before and after BDT, evaluating various parameters each time. The HRM parameters included velopharyngeal contractile integral (VPCI), meso-hypopharyngeal contractile integral (MHPCI), upper esophageal sphincter (UES) relaxation duration, and UES nadir pressure during swallowing (Figure 2). The contractile integral (CI) (mmHg·cm·s) was calculated as amplitude × duration × length of muscular contraction ≥20 mmHg. This parameter serves as an indicator of the “vigor” of pharyngeal contraction. VPCI and MHPCI were used to assess the pharyngeal swallowing pressure [16].

Changes in manometric parameters

Statistical analysis using the Mann-Whitney U test (p > 0.05) revealed no significant changes in pharyngeal contractility (VPCI and MHPCI) (Table 1). However, concerning UES function, UES relaxation duration significantly increased (p < 0.05), and the UES nadir pressure during swallowing significantly decreased (p < 0.01).

**Table 1:** Manometric study before and after balloon dilatation therapy VPCI, velopharyngeal contractile integral; MHPCI, meso-hypopharyngeal contractile integral; UES, upper esophageal sphincter; IQR, interquartile range Statistical analysis performed: the Mann-Whitney U test (p > 0.05).

	Before	After	P value
VPCI (mmHg･cm･s), median [IQR]	22.3 [18.1 - 23.8]	22.2 [20.8 - 25.4]	0.69
MHPCI (mmHg･cm･s), median [IQR]	8.1 [4.6 - 14.2]	5.5 [4.0 - 7.6]	0.55
UES nadir pressure (mmHg), median [IQR]	13.8 [10.6 – 17.4]	4.9 [1.5 – 5.8]	< 0.01
UES relaxation duration (msec), median [IQR]	150 [125 – 160]	400 [315 – 400]	< 0.01

## Discussion

To the best of our knowledge, this is the first study to clarify the immediate effect of BDT on pharyngeal dysphagia with CPD evaluated using HRM pressure dynamics. Most importantly, the immediate effect of BDT on UES pressure was objectively examined using HRM. As an immediate effect, HRM revealed a prolonged UES opening time and a reduction in UES pressure during swallowing in patients with pharyngeal dysphagia with CPD. Lan et al. reported reduced UES residual pressure during swallowing after three weeks of BDT using HRM; however, the immediate effect was unclear. Elucidating the immediate effects of balloon dilatation is crucial in justifying its use before meals or during food-based training. The effectiveness of the BDT would continue during meals and swallowing exercises. However, the duration of the immediate effect on UES dilation remains unclear and warrants further investigation. Furthermore, in this case, the balloon was dilated with air, whereas the previously reported balloon technique used water for dilation [5]. Further studies are needed to examine the immediate effects of other techniques on UES dilatation and balloon types.

In this case, VF revealed improved pharyngeal bolus passage and decreased pharyngeal residues in the pyriform sinus. Furthermore, dysphagia symptoms, including residual pharyngeal sensation and difficulties in bolus passage, improved. The VPCI and MHPCI values for 3 mL thickened liquids in our previous report were 124.3 ± 50.3 and 193.2 ± 34.1, respectively [13]. Regarding the UES function, the UES nadir pressure value was -11.2 mmHg, and the UES relaxation duration value was 520.6 ± 60.0 mmHg, respectively. In this case, pharyngeal contractility was remarkably weak, and BDT improved pharyngeal bolus passage by facilitating UES relaxation, prolonged relaxation and decreased pressure in the UES during swallowing.

Interestingly, this is the first report on the efficacy of BDT in the SMA. The effectiveness of BDT has been reported in patients with pharyngeal dysphagia, including those with brainstem stroke [5,7], neuromuscular diseases [8], and elderly patients [10]. BDT may also be effective in treating dysphagia caused by neurodegenerative diseases. BDT is indicated for dysphagia patients with pharyngeal residue in the pyriform sinus due to impaired UES opening and when compensatory swallowing methods (e.g., head rotation, reclining posture, or texture modification) do not adequately improve pharyngeal bolus passage. BDT should be combined with conventional rehabilitation for impaired UES opening. Further research is needed to expand the indications for BDT in patients with pharyngeal dysphagia with CPD.

## Conclusions

This is the first report to clarify the mechanism behind the immediate effect of BDT on the impaired UES opening, with a focus on pressure dynamics. BDT appears to improve pharyngeal dysphagia in patients with CPD by promoting prolonged relaxation and decreased pressure in the UES during swallowing, suggesting a mechanism for its immediate effectiveness. Furthermore, BDT may demonstrate broader usefulness by potentially improving pharyngeal bolus passage in individuals with SMA and dysphagia; however, further research is warranted to confirm this benefit. The mechanism of the immediate effect of BDT may need validation in other patients with dysphagia, including those with Wallenberg syndrome, in addition to the present case.
